# Causes and consequences of fake transparency/excess information in food claims

**DOI:** 10.1371/journal.pone.0275800

**Published:** 2022-12-08

**Authors:** Susweta Ray, Konstantinos Giannakas

**Affiliations:** Department of Agricultural Economics, University of Nebraska-Lincoln, Lincoln, Nebraska, United States of America; University of Oklahama Norman Campus: The University of Oklahoma, UNITED STATES

## Abstract

This study develops novel multi-stage game-theoretic models of heterogeneous firms and consumers in vertically differentiated food product markets with asymmetric information to analyze the economic causes and market and welfare consequences of excess information/fake transparency in food labeling. Analytical results indicate that the firms’ incentives to adopt the excess information strategy, the Nash equilibrium configuration of firms adopting the strategy, and the market and welfare impacts of excess information are case-specific and dependent on the consumer reaction to excess information, the quality of the firms’ products, the degree of product differentiation between the brand producing firms, and whether the market is covered or not.

## Introduction

When it comes to products with credence attributes (i.e., attributes hidden to consumers even after the purchase and use of a product), consumer purchasing decisions depend largely on the information conveyed through product labels and claims (for a comprehensive review of the literature on labeling see [1]). With growing consumer demands for certain credence product and process attributes of their food (like non-GMO, gluten-free, cholesterol-free products, and organic, low-carbon, cage-free production), it is no surprise that food companies have been aggressively trying to meet the new (and “new”) consumer demands before and better than their rivals.

While most product labels and claims contain information on the key characteristics and differentiating attribute(s) of a product (e.g., organic, cage-free, free-range, dolphin-safe etc.), there have recently been claims on product labels that, even though technically accurate, can be misleading since all relevant products possess the characteristic communicated on the label. Examples of such claims include gluten-free and non-GMO claims on certain bottled water labels, cholesterol-free claim on certain pasta packages, and hormone-free claims of pork products. Even though *all* water is gluten-free and non-GMO, *all* pasta is cholesterol-free, and *all* pork products in the US are hormone-free under federal regulation, many consumers may not have this knowledge.

While the incentives of profit-maximizing firms in adopting such excess information/fake transparency strategy (excess information, hereafter) might appear fairly clear, it is not clear why only some firms in some markets have chosen to adopt this strategy. In addition, different levels of consumer understanding of various claims and degrees of product differentiation between brand-producing firms make the causes and market and welfare consequences of excess information far from obvious.

Despite the prevalence of excess information in food labeling [2], the economic causes and consequences of this strategy have received no attention in the relevant literature. Instead, this literature has focused on the consumer response to the product information provided in the labels, with numerous studies showing that food products with vague claims like “Natural” and “Sustainable” are perceived to be of higher quality by consumers and enjoy an increased willingness to pay even when consumers cannot interpret the meaning of such claims [3, 4]. In general, consumers seem to misinterpret and overestimate the meaning of vague “Natural” claims on food labels [5, 6], and perceive food products with “Free from” claims to be safer, even when the claims are created/made up by the researchers and are not related to a real health concern [7]. Focusing on the reasons behind consumers’ attitudes towards products bearing “redundant” (or excess) information on food labels [8], found that farm versus non-farm background and prior scientific knowledge are key in determining these attitudes.

While the consumer reaction to excess information is certainly important, it is only a part of the causes (and consequences) of the excess information strategy. The objective of this research is to systematically analyze the economic causes and market and welfare consequences of excess information. In particular, this study develops novel game-theoretic models of heterogeneous firms and consumers in quality- (or vertically-) differentiated food product markets with asymmetric information to determine: (a) the conditions under which different quality brand-producing firms will find it optimal to implement this strategy, and (b) the market and welfare impacts of the strategy when it is implemented by different firms (i.e., the impact of excess information on equilibrium quantities and prices of the low- and high-quality products, firm profits and consumer welfare). Different scenarios on the nature of the strategic interaction between the brand-producing firms, the quality of their products and degree of product differentiation, the consumer reaction to excess information, and market coverage (whether the market is covered or not) are considered within this framework.

The rest of the study is organized as follows. The next section presents the game-theoretic framework developed to analyze firms’ excess information and pricing decisions in markets for quality-differentiated products and derives the conditions under which different firms find it optimal to adopt the excess information strategy. The section following determines the changes in prices, quantities, firm profits and consumer welfare under each of these cases. The cases of a covered market and different consumer responses to the excess information strategies of different firms are also analyzed before the final section summarizes and concludes the study.

## Theoretical framework

The relevant strategic interactions among firms considering the excess information strategy in markets for quality-differentiated products are modeled as a two-stage game where: in Stage 1, firms decide whether they will provide excess information on their product labels; and in Stage 2, firms determine strategically their profit-maximizing prices based on the consumer demands for the different quality products. Once the consumer decisions in markets for vertically differentiated products have been analyzed and the demands for the different quality products have been derived, the game is solved using backward induction–the firms’ optimal pricing strategies are considered first, and the solution to the excess information game determines the subgame perfect equilibrium excess information strategies, product prices, and consumer decisions/product choices.

### Consumer decisions in vertically-differentiated product markets

To capture the decisions of consumers differing in the strength of their preference for quality, we consider an uncovered market of vertically differentiated products where consumers have the choice between the low- and high-quality versions of a product, and an alternative good [9]. While consumers agree on the relative quality ranking of these products, they differ in their valuation of the perceived quality difference between the products. Let *θ*∈[0,1] be the attribute that differentiates consumers, with higher values of *θ* indicating a stronger preference for quality. The utility function of the consumer with differentiating attribute *θ* is given by:

*U*_*L*_ = *U*−*p*_*L*_+*αθ*             when a unit of low-quality product is consumed
*U*_*H*_ = *U*−*p*_*H*_+*βθ*             when a unit of high-quality product is consumed
*U*_*A*_ = *U*                  when a unit of the alternative product is consumed

where *U* is a base level of utility associated with the consumption of these products, *p*_*L*_ and *p*_*H*_ are the prices of the low- and high-quality products, respectively, and *α* and *β* are preference parameters/utility enhancement factors associated with the consumption of the low- and high-quality products, respectively. In this context, *U*+*αθ* and *U*+*βθ* give the valuation of (and maximum willingness to pay for) the low- and high-quality product, respectively, for the consumer with differentiating attribute *θ*. The greater the preference parameters *α* and *β*, and/or the greater the differentiating attribute *θ*, the greater the consumer valuation of these products.

To capture the vertical differentiation between the low- and high-quality products, we assume that *β*>*α*, with the difference between *β* and *α* capturing the degree of differentiation between these products and the product (*β*−*α*)*θ* capturing the valuation of the perceived quality difference between the high- and low-quality products of the consumer with differentiating attribute *θ*. To allow for the coexistence of the different quality products in the market we assume that *p*_*H*_>*p*_*L*_, while, to save on notation, the utility associated with the consumption of the alternative product is set equal to the base level of utility. Note that, as done in many studies, the base level of utility could be set equal to zero (in which case the consumers would face the choice between the low-quality product, the high-quality product and not consuming any of these products) without changing the results of this study.

**Fig 1:**
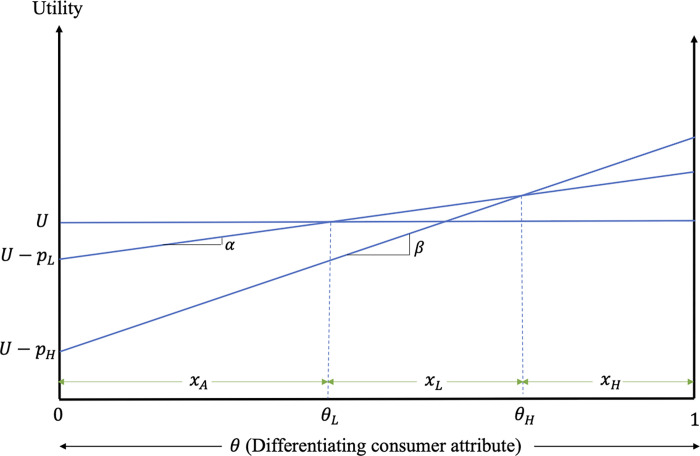
Consumer decisions and welfare.

Consumer decisions are determined by the relative utilities associated with the different products. Fig 1 graphs the utilities associated with the consumption of low-quality, high-quality and alternative products, when the three products coexist in the market.

The consumer with differentiating attribute $\theta_{L}:U_{A}=U_{L}⟹\theta_{L}=\frac{p_{L}}{\alpha }$ is indifferent between the alternative and the low-quality products, while the consumer with $\theta_{H}:U_{L}=U_{H}⟹\theta_{H}=\frac{p_{H}-p_{L}}{\beta -\alpha }$ is indifferent between the low- and high-quality products. Consumers with stronger preference for quality (i.e., consumers with *θ*∈(*θ*_*H*_, 1]) prefer the high-quality product, consumers with *θ*∈(*θ*_*L*_, *θ*_*H*_) prefer the low-quality product, while consumers with *θ*∈[0, *θ*_*L*_) prefer the alternative product. Assuming consumers are uniformly distributed with respect to their differentiating attribute and normalizing their mass to unity, we can derive the demands for low- and high-quality products as a function of the product prices, *p*_*L*_ and *p*_*H*_, and preference parameters, *α* and *β*,

$$x_{L}=\theta_{H}-\theta_{L}=\frac{\alpha p_{H}-\beta p_{L}}{\alpha (\beta -\alpha )}$$ (1)

$$x_{H}=1-\theta_{H}=\frac{\beta -\alpha +p_{L}-p_{H}}{\beta -\alpha }$$ (2)

### Stage 2: Firm pricing decisions

At the supply side of this vertically-differentiated market, there are two brand-producing firms, termed low-quality firm and high-quality firm, producing the low-and high-quality product, respectively. At the second stage of the game, the low- and high-quality firms are involved in a price competition and seek to maximize their profits. For simplicity and without loss of generality (as the firms’ product quality choices are exogenous in our analysis), we normalize the firms’ marginal costs of production to zero.

Given the consumer demands for the two products in Eqs (1) and (2), the low-quality firm’s profit maximization problem can be expressed as:

$$\underset{p_{L}}{\max}\;\Pi_{L}=p_{L}x_{L}=p_{L}\left[\frac{\alpha p_{H}-\beta p_{L}}{\alpha (\beta -\alpha )}\right]=\frac{\alpha p_{H}p_{L}}{\alpha (\beta -\alpha )}-\frac{\beta p_{L}^{2}}{\alpha (\beta -\alpha )}$$ (3)

From the first order condition for a maximum, we can derive the best response function of this firm as:

$$p_{L}=\frac{\alpha p_{H}}{2\beta }$$ (4)

Similarly, the optimization problem of the high-quality firm is:

$$\underset{p_{H}}{\max}\;\Pi_{H}=p_{H}x_{H}=p_{H}\left(\frac{\beta -\alpha +p_{L}-p_{H}}{\beta -\alpha }\right)=\frac{(\beta -\alpha +p_{L})p_{H}}{\beta -\alpha }-\frac{p_{H}^{2}}{\beta -\alpha }$$ (5)

and its first order condition yields the best response function of the high-quality firm as:

$$p_{H}=\frac{\beta -\alpha +p_{L}}{2}$$ (6)

Solving Eqs (4) and (6) simultaneously, we get the Nash equilibrium prices as:

$$p_{L}^{*}=\frac{\alpha (\beta -\alpha )}{4\beta -\alpha }$$ (7)

$$p_{H}^{*}=\frac{2\beta (\beta -\alpha )}{4\beta -\alpha }$$ (8)

Substituting $p_{L}^{*}$ and $p_{H}^{*}$ into the Eqs (1) and (2), we can derive the equilibrium quantities of the low- and high-quality products as:

$$x_{L}^{*}=\frac{\beta }{4\beta -\alpha }$$ (9)

$$x_{H}^{*}=\frac{2\beta }{4\beta -\alpha }$$ (10)

Using the equilibrium prices and quantities, we can derive the firms’ profits as functions of the preference parameters associated with the consumption of the low- and high-quality products, *α* and *β*, as:

$$\Pi_{L}=p_{L}^{*}x_{L}^{*}=\left[\frac{\alpha (\beta -\alpha )}{4\beta -\alpha }\right]\left(\frac{\beta }{4\beta -\alpha }\right)=\frac{\alpha \beta (\beta -\alpha )}{{\left(4\beta -\alpha \right)}^{2}}$$ (11)

$$\Pi_{H}=p_{H}^{*}x_{H}^{*}=\left[\frac{2\beta (\beta -\alpha )}{4\beta -\alpha }\right]\left(\frac{2\beta }{4\beta -\alpha }\right)=\frac{4\beta^{2}(\beta -\alpha )}{{\left(4\beta -\alpha \right)}^{2}}$$ (12)

### Stage 1: Excess information decisions

When brand-producing firms introduce excess information in their food product labels (e.g., “gluten-free” or “non-GMO” claim in bottled water or salt, “cholesterol-free” claim in pasta, “hormone-free” claim in pork products etc.), this information can impact the consumer valuation of these products in a positive or negative way depending on whether consumers are aware of the nature of this information or not. For example, if consumers know that all bottled water is “non-GMO”, the presence of such label can be perceived as being disingenuous and manipulative and result in reduced consumer valuation of the brand bearing this label. On the other hand, if consumers are not aware that all pasta products are “cholesterol-free” or that all pork products in the US are “hormone-free”, for instance, the presence of such label on a product can result in increased consumer valuation of this product. In either case, the magnitude of the consumer response to excess information depends on how much consumers care about the quality characteristics of the relevant products [10]. The stronger the consumer preference for quality (captured by the value of the consumer differentiating attribute *θ* in our model), the stronger their (positive or negative) response to excess information can be expected to be.

In this context, by introducing excess information on the labels of their products, firms can affect the consumer valuation of their products (and their profits). When the consumer reaction to excess information is positive (negative), the introduction of excess information by the high-quality firm increases (decreases) the consumer valuation of its product, *β*. As shown by Eqs (11) and (12), the increase (decrease) in *β* causes the profits of both high- and low-quality firms to increase (decrease), as $\frac{\partial \Pi_{H}}{\partial \beta }>0$ and $\frac{\partial \Pi_{L}}{\partial \beta }>0$. On the other hand, when the consumer reaction to excess information is positive (negative) and the low-quality firm adopts the strategy, that increases (decreases) the consumer valuation of the low-quality product, *α*, which, in turn, increases (decreases) the profits of the low-quality firm as long as the low- and high-quality products have a degree of differentiation where $\alpha <\frac{4}{7}\beta (\alpha >\frac{4}{7}\beta )$, and decreases (increases) the profits of the high-quality firm, i.e., $\frac{\partial \Pi_{L}}{\partial \alpha }≶0$ when $\alpha ≷\frac{4}{7}\beta$ and $\frac{\partial \Pi_{H}}{\partial \alpha }<0$.

Π_*L*_ falls with an increase in *α* when the products are not highly differentiated (i.e., $\alpha >\frac{4}{7}\beta$), as the benefits from the increased consumer demand for the low-quality product, $x_{L}^{*}$, are outweighed by the losses from the reduction in price $p_{L}^{*}$ due to reduced product differentiation. On the other hand, the profits of the low-quality firm always increase with an increase in the consumer valuation/strength of the consumer preference for the high-quality product, *β* (i.e., $\frac{{\partial \Pi }_{L}}{\partial \beta }>0$), as an increase in *β* increases the differentiation between the low- and high-quality products. Similarly, Eq (12) reveals that the profits of the high-quality firm increase with an increase in the differentiation between the low- and high-quality products (caused either by an increase in *β* or a reduction in *α*)–i.e., $\frac{{\partial \Pi }_{H}}{\partial \beta }>0$ and $\frac{{\partial \Pi }_{H}}{\partial \alpha }<0$. Note that the analysis can be modified to account for impacts of excess information on the consumer valuation of the substitute product (like the provision of excess information by the high-quality firm increasing *β and* reducing *α*; see [11]) without affecting the qualitative results of this study.

**Result 1:** While the high-quality firm always prefers a higher consumer valuation of its product, the preference of the low-quality firm depends on the degree of differentiation between the low- and high-quality products. When the low- and high-quality products are (are not) highly differentiated, the low-quality firm prefers a higher (lower) consumer valuation of its product.

As the introduction of excess information on a product label can increase or reduce the consumer valuation of the product, which, along with the degree of product differentiation, can influence firms’ profits, there are four different cases that can emerge and are considered in this study. These cases correspond to different consumer reactions to excess information and degrees of product differentiation in the market. For each of these cases, we consider the decision of the low- and high-quality firms to strategically provide (or not) excess information knowing how this decision will affect their market profits in Stage 2 (given by Eqs (11) and (12)). Sequential decision making on the adoption of the excess information strategy at the first stage of the game is also considered and shown to not affect the results of our study (see S1 Appendix).

When the two firms decide strategically (i.e., simultaneously) whether to adopt the excess information strategy or not, the payoff matrix of this finite game (depicting the strategies of the two firms and the payoffs that result from different strategy profiles/combinations of strategies) can be represented as:

**Table:** 

		Firm L	
		*E*	*N*
Firm H	*E*	$\Pi_{H}^{E,E},\Pi_{L}^{E,E}$	$\Pi_{H}^{E,N},\Pi_{L}^{E,N}$
	*N*	$\Pi_{H}^{N,E},\Pi_{L}^{N,E}$	$\Pi_{H}^{N,N},\Pi_{L}^{N,N}$

where *E* and *N* denote the strategies of adopting excess information and not adopting, respectively. The payoff $\Pi_{H}^{i,j}(\Pi_{L}^{i,j})$ indicates the profit of the high-quality (low-quality) firm when the high-and low-quality firms choose strategy *i* and *j*, respectively, where *i*, *j*∈{*E*, *N*}.

### Case I: When the consumer reaction to excess information is positive and the low- and high-quality products are highly differentiated

When the consumer reaction to excess information is positive and the difference between the two products is such that $\alpha <\frac{4}{7}\beta$, the profits of the low-quality firm are greater when it adopts the strategy compared to when it does not (as, in this case, $\frac{\partial \Pi_{L}}{\partial \alpha }>0$). Given that the adoption of the strategy, in this case, increases the consumer valuation of the relevant product and $\frac{\partial \Pi_{H}}{\partial \beta }>0$, the best response of the high-quality firm is to also adopt the strategy irrespective of the low-quality firm’s decision. As the provision of excess information is a dominant strategy for both firms, (*E*, *E*) is the strategy profile that none of the firms has incentive to deviate from, making the provision of excess information by both firms (*E*, *E*) the unique Nash equilibrium in this case. The payoff matrix below depicts, in bold, the best responses of each firm to the strategies of its rival as well as the Nash equilibrium of this strategic interaction (where each firm’s strategy is the best response to the other firm’s equilibrium strategy).

**Table:** 

		Firm L	
		*E*	*N*
Firm H	*E*	$\Pi_{H}^{E,E*},\Pi_{L}^{E,E*}$	$\Pi_{H}^{E,N*},\Pi_{L}^{E,N}$
	*N*	$\Pi_{H}^{N,E},\Pi_{L}^{N,E*}$	$\Pi_{H}^{N,N},\Pi_{L}^{N,N}$

**Result 2:** When the consumer reaction to excess information is positive and products are highly differentiated, both high- and low-quality firms will adopt the excess information strategy.

### Case II: When the consumer reaction to excess information is positive and the low- and high- quality products are not highly differentiated

When the consumer reaction to excess information is positive but the two products are not highly differentiated (i.e., $\alpha >\frac{4}{7}\beta$), an increase in *α* due to excess information provision reduces the profit of the low-quality firm (as $\frac{\partial \Pi_{L}}{\partial \alpha }<0$ in this case) and the firm does not adopt the strategy. The high-quality firm, on the other hand, finds it optimal to adopt the strategy no matter the strategy of the low-quality firm (as $\frac{\partial \Pi_{H}}{\partial \beta }>0$). Therefore, in this case (*E*, *N*) is the unique Nash equilibrium with only the high-quality firm adopting the excess information strategy (see payoff matrix below).

**Table:** 

		Firm L	
		*E*	*N*
Firm H	*E*	$\Pi_{H}^{E,E*},\Pi_{L}^{E,E}$	$\Pi_{H}^{E,N*},\Pi_{L}^{E,N*}$
	*N*	$\Pi_{H}^{N,E},\Pi_{L}^{N,E}$	$\Pi_{H}^{N,N},\Pi_{L}^{N,N*}$

**Result 3:** When the consumer reaction to excess information is positive and products are not highly differentiated, only the high-quality firm will adopt the excess information strategy.

### Case III: When the consumer reaction to excess information is negative and the low- and high-quality products are highly differentiated

When the consumer reaction to excess information is negative and the low- and high-quality products are highly differentiated (i.e., $\alpha <\frac{4}{7}\beta$), the profits of the low-quality firm when it does not adopt the strategy are higher than when it adopts the strategy (due to the lower valuation of the product that contains excess information and $\frac{\partial \Pi_{L}}{\partial \alpha }>0$). Thus, not providing excess information is the dominant strategy for the low-quality firm. For the same reason, the best response of the high-quality firm is also not to adopt the strategy no matter what the strategy of the low-quality firm is. Therefore, when the consumer valuation of excess information is negative and the two products are highly differentiated, then (*N*, *N*) is the Nash equilibrium and there will be no excess information in the market (see payoff matrix below).

**Table:** 

		Firm L	
		*E*	*N*
Firm H	*E*	$\Pi_{H}^{E,E},\Pi_{L}^{E,E}$	$\Pi_{H}^{E,N},\Pi_{L}^{E,N*}$
	*N*	$\Pi_{H}^{N,E*},\Pi_{L}^{N,E}$	$\Pi_{H}^{N,N*},\Pi_{L}^{N,N}*$

**Result 4:** When the consumer reaction to excess information is negative and products are highly differentiated, firms will not adopt the excess information strategy.

### Case IV: The consumer reaction to excess information is negative and the low- and high-quality products are not highly differentiated

When the consumer reaction to excess information is negative but the low- and high-quality products are not highly differentiated (i.e., $\alpha >\frac{4}{7}\beta$), the low-quality firm finds it optimal to provide excess information as the strategy increases the degree of product differentiation/perceived quality difference between the two products and the profits of the low-quality firm. The high-quality firm, on the other hand, does not adopt the strategy as a decrease in *β* reduces its profits. Therefore, under this scenario (*N*, *E*) is the unique Nash equilibrium with only the low-quality firm adopting the excess information strategy (see payoff matrix below).

**Table:** 

		Firm L	
		*E*	*N*
Firm H	*E*	$\Pi_{H}^{E,E},\Pi_{L}^{E,E*}$	$\Pi_{H}^{E,N},\Pi_{L}^{E,N}$
	*N*	$\Pi_{H}^{N,E*},\Pi_{L}^{N,E*}$	$\Pi_{H}^{N,N*},\Pi_{L}^{N,N}$

**Result 5:** When the consumer reaction to excess information is negative and products are not highly differentiated, only the low-quality firm will adopt the excess information strategy.

### Market and welfare impacts of excess information

After having determined the conditions under which different strategy profiles constitute Nash equilibria in the excess information games between the low- and high-quality firms, we focus next on the market and welfare effects of excess information–i.e., the impacts of the strategy on the prices and quantities of the low- and high-quality products, firm profits and consumer welfare–under each of these equilibria.

### Case I: Both low- and high-quality firms provide excess information (the consumer reaction to excess information is positive and the low- and high-quality products are highly differentiated)

When the consumer reaction to excess information is positive and both low- and high-quality firms adopt the strategy, excess information increases the consumer valuation of low- and high-quality products to *α*′ and *β*′, respectively, where *α*′ = *α*+*ε* and *β*′ = *β*+*ε* with *ε*>0 being the value consumers place on the product attribute communicated through the provision of excess information (like the “cholesterol-free” attribute of pasta or the “hormone-free” attribute of pork). The equilibrium prices of these products in the second stage of the game (derived by substituting *α*′ and *β*′ for *α* and *β*, respectively, in Eqs (7) and (8)) are given by:

$${p_{L}^{\prime }}^{*}=\frac{\alpha^{\prime }(\beta \prime -\alpha \prime )}{4\beta \prime -\alpha \prime }=\frac{\left(\alpha +\varepsilon )(\beta -\alpha \right)}{3\varepsilon +4\beta -\alpha }$$ (13)

$${p_{H}^{\prime }}^{*}=\frac{2\beta \prime (\beta \prime -\alpha \prime )}{4\beta \prime -\alpha \prime }=\frac{2(\beta +\varepsilon )(\beta -\alpha )}{3\varepsilon +4\beta -\alpha }$$ (14)

The equilibrium prices are higher when both firms adopt the strategy compared to the no excess information scenario, with the increase in the price of the low-quality product being greater than that of high-quality product.

The equilibrium quantities of the low- and high-quality products in this case are:

$${x_{L}^{\prime }}^{*}=\frac{\beta^{\prime }}{4\beta \prime -\alpha \prime }=\frac{\beta +\varepsilon }{3\varepsilon +4\beta -\alpha }$$ (15)

$${x_{H}^{\prime }}^{*}=\frac{2\beta \prime }{4\beta \prime -\alpha \prime }=\frac{2(\beta +\varepsilon )}{3\varepsilon +4\beta -\alpha }$$ (16)

The profits of the firms are given by:

$$\Pi_{L}^{E}={p_{L}^{\prime }}^{*}{x_{L}^{\prime }}^{*}=\frac{\alpha^{\prime }\beta \prime (\beta \prime -\alpha \prime )}{{\left(4\beta \prime -\alpha \prime \right)}^{2}}=\frac{\left(\alpha +\varepsilon )(\beta +\varepsilon )(\beta -\alpha \right)}{{\left(3\varepsilon +4\beta -\alpha \right)}^{2}}$$ (17)

$$\Pi_{H}^{E}={p_{H}^{\prime }}^{*}{x_{H}^{\prime }}^{*}=\frac{4{\beta \prime }^{2}(\beta \prime -\alpha \prime )}{{\left(4\beta \prime -\alpha \prime \right)}^{2}}=\frac{{4(\beta +\varepsilon )}^{2}(\beta -\alpha )}{{\left(3\varepsilon +4\beta -\alpha \right)}^{2}}$$ (18)

and are higher than those under no excess information.

**Fig 2:**
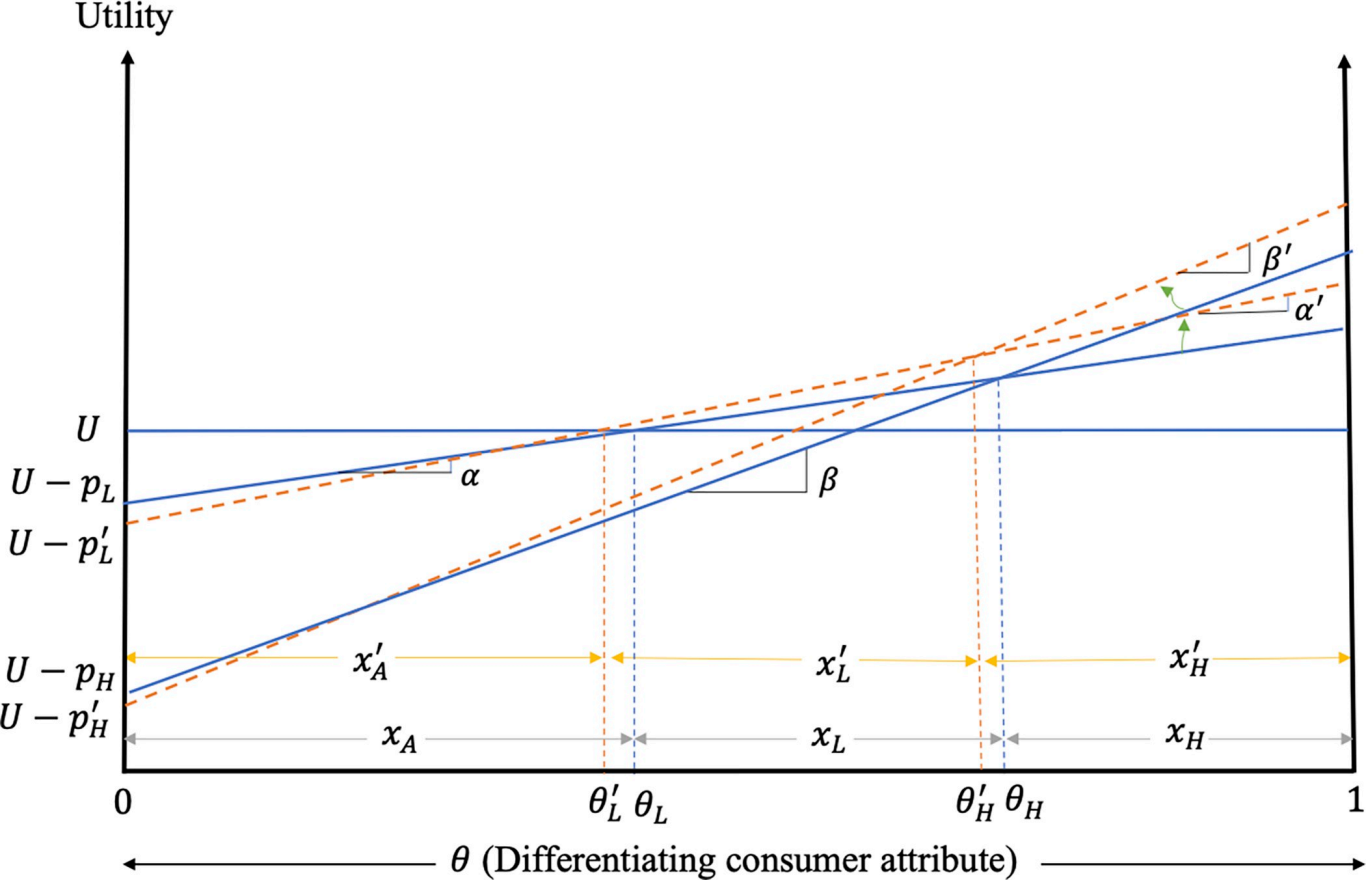
Impacts of excess information on consumer decisions and welfare when both low- and high-quality firms provide excess information.

Fig 2 graphs the impacts of excess information on consumer decisions and welfare when both low- and high-quality firms adopt the strategy.

Fig 2 shows that consumers with $\theta \in (\theta_{L}^{\prime },\theta_{L}]$ switch from the alternative to the low-quality product (due to the increased consumer valuation of the low-quality product relative to the alternative product), while consumers with $\theta \in [\theta_{H}^{\prime }{,\theta }_{H})$ switch from the low-quality product to its high-quality counterpart (due to the greater increase in the price of the low-quality product) after the introduction of excess information. The outcome is increased market shares of both the low- and high-quality products (compare *x*_*L*_ to $x_{L}^{\prime }$ and *x*_*H*_ to $x_{H}^{\prime }$ in Fig 2).

The change in welfare of the consumers of low-quality product in this case is given by $\Delta W_{L}^{c}=\int_{\theta_{L}^{\prime }}^{\theta_{L}}(U_{L}^{\prime }-U_{A})d\theta +\int_{\theta_{L}}^{\theta_{H}^{\prime }}(U_{L}^{\prime }-U_{L})d\theta$, while the welfare change of the consumers of the high-quality product is given by $\Delta W_{H}^{c}=\int_{\theta_{H}^{\prime }}^{\theta_{H}}(U_{H}^{\prime }-U_{L})d\theta +\int_{\theta_{H}}^{1}(U_{H}^{\prime }-U_{H})d\theta$, where $U_{L}^{\prime }$ and $U_{H}^{\prime }$ are the perceived utilities associated with the consumption of the low- and high-quality product, respectively, in the presence of excess information. As the consumer valuation of excess information is positive in this case, all consumers of low- and high-quality products realize welfare gains, with those gains increasing with the consumer valuation of quality (see Fig 2).

The results of Case I can be summarized as follows.

**Result 6:** When the consumer reaction to excess information is positive, products are highly differentiated and both low- and high-quality firms adopt the excess information strategy, excess information increases the market shares of both the low- and high-quality products. Prices of both products also increase and so do the profits of the different quality firms. Consumers of low- and high-quality products benefit from the excess information with those welfare gains increasing with the consumer valuation of quality. All interest groups gain in this case as firms are able to costlessly provide information that consumers value.

### Case II: Only the high-quality firm provides excess information (consumer reaction to excess information is positive and the low- and high-quality products are not highly differentiated)

When the consumer valuation of excess information is positive and only the high-quality firm adopts the strategy, the consumer valuation of the high-quality product increases to *β*′ (= *β*+*ε*), and the equilibrium prices of low- and high-quality products in the second stage of the game are given by:

$${p_{L}^{\prime }}^{*}=\frac{\alpha (\beta^{\prime }-\alpha )}{4\beta^{\prime }-\alpha }=\frac{\alpha (\beta +\varepsilon -\alpha )}{4\varepsilon +4\beta -\alpha }$$ (19)

$${p_{H}^{\prime }}^{*}=\frac{2\beta^{\prime }(\beta^{\prime }-\alpha )}{4\beta^{\prime }-\alpha }=\frac{2(\beta +\varepsilon )(\beta +\varepsilon -\alpha )}{4\varepsilon +4\beta -\alpha }$$ (20)

As, under this scenario, *β*′>*β*, the equilibrium prices of the low- and high-quality products are higher than those under no excess information.

The equilibrium quantities of low- and high-quality products in this case are:

$${x_{L}^{\prime }}^{*}=\frac{\beta^{\prime }}{4\beta^{\prime }-\alpha }=\frac{\beta +\varepsilon }{4\varepsilon +4\beta -\alpha }$$ (21)

$${x_{H}^{\prime }}^{*}=\frac{2\beta^{\prime }}{4\beta^{\prime }-\alpha }=\frac{2(\beta +\varepsilon )}{4\varepsilon +4\beta -\alpha }$$ (22)

and the firm profits are given by:

$$\Pi_{L}^{N}=p_{L}^{*}{x_{L}^{\prime }}^{*}=\frac{\alpha \beta^{\prime }(\beta^{\prime }-\alpha )}{{\left(4\beta^{\prime }-\alpha \right)}^{2}}=\frac{\alpha (\beta +\varepsilon )(\beta +\varepsilon -\alpha )}{{\left(4\varepsilon +4\beta -\alpha \right)}^{2}}$$ (23)

$$\Pi_{H}^{E}=p_{H}^{*}{x_{H}^{\prime }}^{*}=\frac{4{\beta^{\prime }}^{2}(\beta^{\prime }-\alpha )}{{\left(4\beta^{\prime }-\alpha \right)}^{2}}=\frac{4{\left(\beta +\varepsilon \right)}^{2}(\beta +\varepsilon -\alpha )}{{\left(4\varepsilon +4\beta -\alpha \right)}^{2}}$$ (24)

The increased product differentiation that emerges when only the high-quality firm adopts the strategy results in the profits of both low- and high-quality firms being higher than their profits under the benchmark case of no excess information as $\frac{\partial \Pi_{H}}{\partial \beta }>0$ and $\frac{\partial \Pi_{L}}{\partial \beta }>0$.

**Fig 3:**
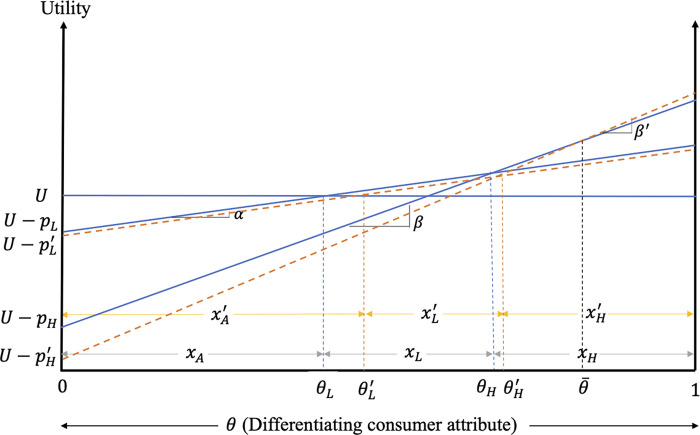
Impacts of excess information on consumer decisions and welfare when only the high-quality firm provides excess information.

The impacts of excess information on consumer decisions and welfare when only the high-quality firm adopts the strategy are graphed in Fig 3.

As shown in this figure, the positive consumer reaction to excess information results in welfare gains for high-quality product consumers with stronger preference for quality (i.e., consumers with $\theta \in (\bar{\theta },1]$ in Fig 3). Consumers of the high-quality product with weaker preference for quality (i.e., those with $\theta \in [\theta_{H},\bar{\theta })$) lose as the benefits from the increased valuation of this product are outweighed by the loses from its higher price. In fact, the price increase of this product exceeds that of its low-quality counterpart, resulting in reduced market share of the high-quality product as consumers with $\theta \in (\theta_{H},\theta_{H}^{\prime }]$ find it optimal to switch to the low-quality product. Despite the entry of previous high-quality product consumers, the market share of the low-quality product also falls in this case, as its increased price results in consumers with $\theta \in (\theta_{L},\theta_{L}^{\prime }]$ switching to the alternative product (with the size of this group exceeding that switching from the high-quality product). In addition to reducing the market share of, and demand for the low-quality product, the increased price of this product results in welfare losses for the consumers of low-quality product. The welfare changes for the consumers of the low- and high-quality products are given by $\Delta W_{L}^{c}=\int_{\theta_{L}}^{\theta_{L}^{\prime }}(U_{A}-U_{L})d\theta +\int_{\theta_{L}^{\prime }}^{\theta_{H}}(U_{L}^{\prime }-U_{L})d\theta$ and $\Delta W_{H}^{c}=\int_{\theta_{H}}^{\theta_{H}^{\prime }}(U_{L}^{\prime }-U_{H})d\theta +\int_{\theta_{H}^{\prime }}^{1}(U_{H}^{\prime }-U_{H})d\theta$, respectively, while the results of Case II can be summarized as follows.

**Result 7:** When the consumer reaction to excess information is positive, products are not highly differentiated and only the high-quality firm adopts the excess information strategy, excess information increases the prices and reduces the market shares of both the low- and the high-quality products. The profits of the low- and high-quality firms increase due to the increased product differentiation that emerges when only the high-quality firm provides excess information. Consumers of the high-quality product with relatively higher valuation of quality gain, while high-quality product consumers with lower valuation of quality and consumers of the low-quality product lose due to the higher product prices.

### Case III: No firm provides excess information (the consumer reaction to excess information is negative and the low- and high-quality products are highly differentiated)

As no firm finds it optimal to provide excess information in this case, the equilibrium prices, quantities and interest group welfare remain unaffected and are those given by Eqs (7)–(12).

### Case IV: Only the low-quality firm provides excess information (consumer reaction to excess information is negative and the low- and high-quality products are not highly differentiated)

When consumer reaction to excess information is negative and only the low-quality firm adopts the strategy, the consumer valuation of the low-quality product falls to *α*′′, where *α*′′ = *α*−*δ* with *δ*>0. The equilibrium prices of the low- and high-quality products in the second stage of the game are, then:

$$p_{L}^{*}=\frac{\alpha^{\prime \prime }(\beta -\alpha^{\prime \prime })}{4\beta -\alpha^{\prime \prime }}=\frac{\left(\alpha -\delta )(\beta -\alpha +\delta \right)}{4\beta -\alpha +\delta }$$ (25)

$$p_{H}^{*}=\frac{2\beta (\beta -\alpha^{\prime \prime })}{4\beta -\alpha^{\prime \prime }}=\frac{2\beta (\beta -\alpha +\delta )}{4\beta -\alpha +\delta }$$ (26)

and are higher than those under no excess information.

The equilibrium quantities of the low- and high-quality products are:

$${x_{L}^{\prime }}^{*}=\frac{\beta }{4\beta -\alpha^{\prime \prime }}=\frac{\beta }{4\beta -\alpha +\delta }$$ (27)

$${x_{H}^{\prime }}^{*}=\frac{2\beta }{4\beta -\alpha^{\prime \prime }}=\frac{2\beta }{4\beta -\alpha +\delta }$$ (28)

and the profits of the low- and high-quality firms at this stage are, then:

$$\Pi_{L}^{E}=p_{L}^{*}{x_{L}^{\prime }}^{*}=\frac{\alpha^{\prime \prime }\beta (\beta -\alpha^{\prime \prime })}{{\left(4\beta -\alpha^{\prime \prime }\right)}^{2}}=\frac{\left(\alpha -\delta )\beta (\beta -\alpha +\delta \right)}{{\left(4\beta -\alpha +\delta \right)}^{2}}$$ (29)

$$\Pi_{H}^{N}=p_{H}^{*}{x_{H}^{\prime }}^{*}=\frac{4\beta^{2}(\beta -\alpha^{\prime \prime })}{{\left(4\beta -\alpha^{\prime \prime }\right)}^{2}}=\frac{4\beta^{2}(\beta -\alpha +\delta )}{{\left(4\beta -\alpha +\delta \right)}^{2}}$$ (30)

The profits of both low- and high-quality firms are higher than those under no excess information due to the increased product differentiation that emerges when the low-quality firm adopts the strategy and *α* is reduced (due to the negative consumer reaction to excess information).

**Fig 4:**
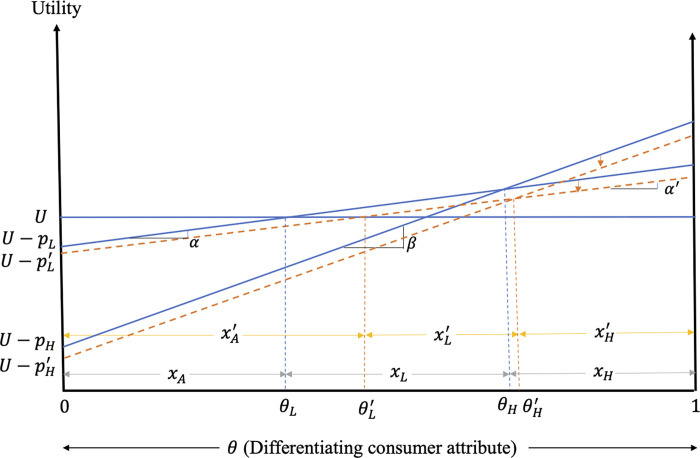
Impacts of excess information on consumer decisions and welfare when only the low-quality firm provides excess information.

The impacts of excess information on consumer purchasing decisions and welfare when only the low-quality firms adopts the strategy are graphed in Fig 4. Fig 4 shows that, due to the negative consumer reaction to excess information and higher price of the low-quality product, the demand for the low-quality product falls from *x*_*L*_ to $x_{L}^{\prime }$ as consumers with $\theta \in (\theta_{L},\theta_{L}^{\prime }]$ switch from the low-quality product to the alternative product. On the other hand, due to the greater increase in the price of the high-quality product, consumers with $\theta \in (\theta_{H},\theta_{H}^{\prime }]$ switch to the low-quality product.

The changes in the welfare of the consumers of low-quality product are given by $\Delta W_{L}^{c}=\int_{\theta_{L}}^{\theta_{L}^{\prime }}(U_{A}-U_{L})d\theta +\int_{\theta_{L}^{\prime }}^{\theta_{H}}(U_{L}^{\prime }-U_{L})d\theta$. The welfare of consumers of the low-quality product falls due to the reduced valuation and higher price of this product. Regarding the changes in the welfare of the high-quality product consumers, they are given by $\Delta W_{H}^{c}=\int_{\theta_{H}}^{\theta_{H}^{\prime }}(U_{L}^{\prime }-U_{H})d\theta +\int_{\theta_{H}^{\prime }}^{1}(U_{H}^{\prime }-U_{H})d\theta$. As the price of the high-quality product increases, the consumers of high-quality product experience losses in welfare even though the product they consume contains no excess information.

**Result 8:** When the consumer reaction to excess information is negative, the products are not highly differentiated and only the low-quality firm adopts the strategy, the equilibrium prices of low- and high-quality products increase due to the increase in the degree of product differentiation. The higher price and lower consumer valuation of the low-quality product results in reduced market share and welfare losses for the consumers of this product. Among the consumers of low-quality product, those with weaker preference for quality switch to the alternative product. The market share of the high-quality product and the welfare of consumers of this product fall due to the price increase. The higher price results in the consumers of high-quality product with weaker preference for quality switching to low-quality product. Both low- and high-quality firms earn higher profits compared to the no excess information case.

Overall, the impacts of excess information on different consumers and brand producing firms under the different cases considered in this study indicate that, under positive consumer reaction to excess information, the introduction of excess information on food claims by both firms benefits the consumers of the low- and high-quality products the most. When the consumer reaction to excess information is negative, both consumer groups prefer that firms do not adopt the strategy. The high-quality firm profits are the highest when it is the only one adopting the strategy, while the low-quality firm’s profits are the highest when the consumer reaction to excess information is positive and both firms adopt the strategy.

### Extension of the analysis I: Different consumer reactions to firms’ excess information strategies

The previous analysis assumes that consumers have the same response to the firms’ excess information strategies. While this is realistic when firms employ the same excess information strategy (like labeling water as “non-GMO” or pasta as “cholesterol-free”), consumer responses to the provision of excess information might differ when firms employ different excess information strategies (e.g., one labeling its water as “non-GMO” while the other labeling it as “gluten-free”). This section analyzes the cases where consumer reactions to the excess information provided by the low- and high-quality firms differ.

### Case a: When the consumer reaction to excess information provided by the low-quality firm is negative, while the reaction to excess information provided by the high-quality firm is positive

Under this scenario, when the low- and high-quality products are highly differentiated, the high-quality firm’s dominant strategy is to provide excess information (as $\frac{\partial \Pi_{H}}{\partial \beta }>0$), while the low-quality firm does not adopt the strategy (as $\frac{\partial \Pi_{L}}{\partial \alpha }>0$ when $\alpha <\frac{4}{7}\beta$). Thus, (*E*, *N*) is the unique Nash equilibrium in this case.

On the other hand, when the products are not highly differentiated, the low-quality firm’s best response is to adopt the strategy (as $\frac{\partial \Pi_{L}}{\partial \alpha }<0$ when $\alpha >\frac{4}{7}\beta$). Adoption of the strategy remains the dominant strategy of the high-quality firm (as $\frac{\partial \Pi_{H}}{\partial \beta }>0$) making (*E*, *E*) the unique Nash equilibrium.

### Case b: When the consumer reaction to excess information provided by the low-quality firm is positive, while the reaction to excess information provided by the high-quality firm is negative

In this case, when the low- and high-quality products are highly differentiated, the high-quality firm’s dominant strategy is to not provide excess information (as $\frac{\partial \Pi_{H}}{\partial \beta }>0$), while the low-quality firm’s dominant strategy is to adopt the strategy (as $\frac{\partial \Pi_{L}}{\partial \alpha }>0$). Therefore, (*N*, *E*) is the unique Nash equilibrium.

When the low- and high-quality products are not highly differentiated, then neither firm adopts the strategy (as $\frac{\partial \Pi_{H}}{\partial \beta }>0$ and $\frac{\partial \Pi_{L}}{\partial \alpha }<0$) making (*N*, *N*) the unique Nash equilibrium in this case.

## Extension of the analysis II: Covered market

While the previous analysis focuses on the causes and consequences of excess information in an uncovered market, this part of the analysis considers the incidence of excess information in a covered market (defined as a market in which all consumers buy either the low- or the high-quality version of the product in question). In particular, following the game-theoretic structure of the previous section, we determine the causes and market and welfare consequences of excess information in a covered market.

### Consumer decisions in a covered vertically-differentiated market

**Fig 5:**
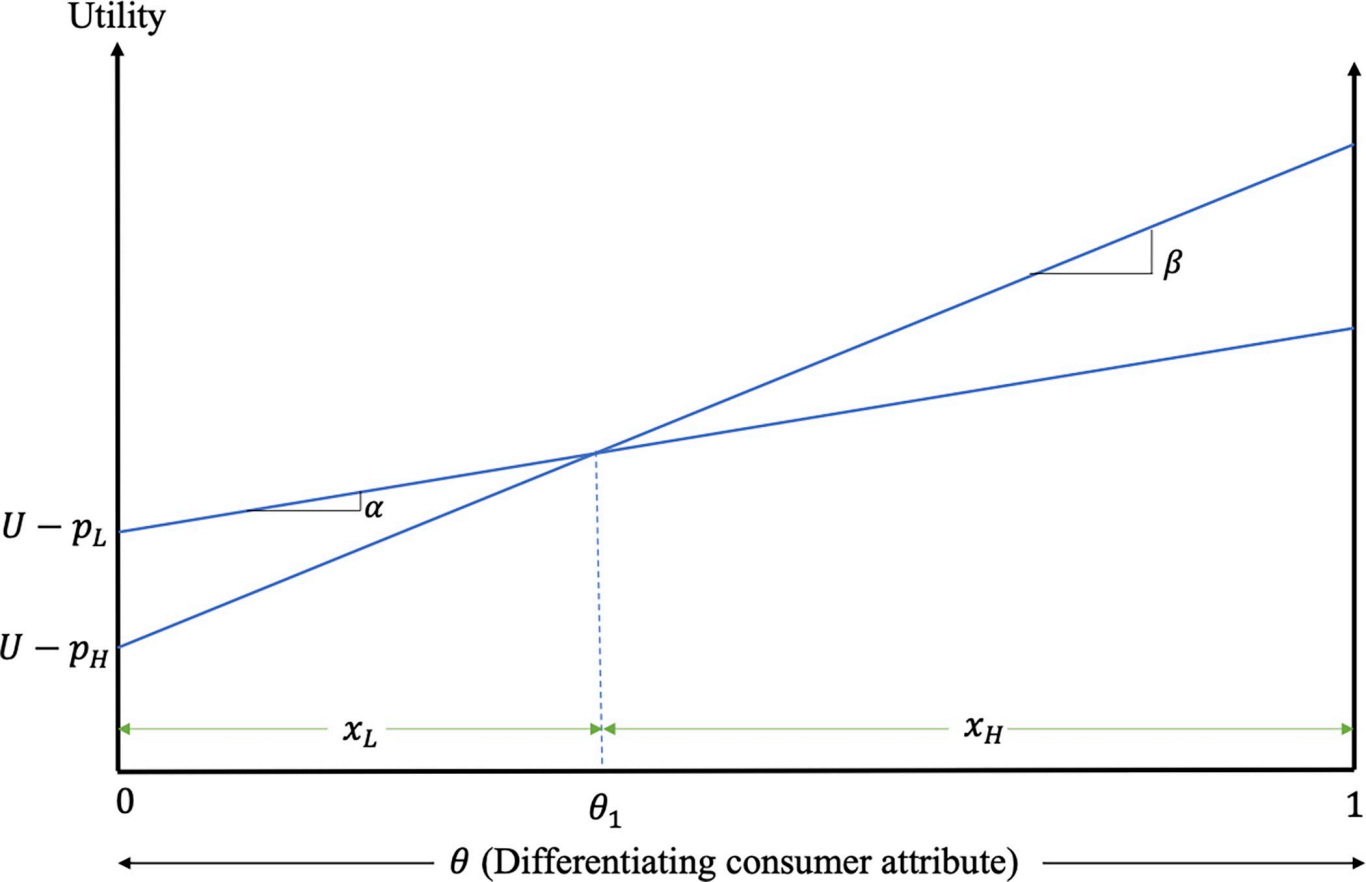
Consumer decisions and welfare in a covered market.

In a covered market, consumers can consume either the low-quality or the high-quality versions of the product in question (unlike the uncovered market, consumers do not have the option of buying an alternative product). Assuming the utilities associated with the consumption of low- and high-quality products remain unchanged, they can be graphically represented as shown in Fig 5.

Following the process established earlier, the demands for the low- and high-quality products are:

$$x_{L}=\frac{p_{H}-p_{L}}{\beta -\alpha }$$ (31)

$$x_{H}=\frac{\beta -\alpha +p_{L}-p_{H}}{\beta -\alpha }$$ (32)

### Stage 2: Firm pricing decisions in a covered market

In the second stage of the game, the firms choose prices to maximize their profits, which results in equilibrium prices:

$$p_{L}^{*}=\frac{\beta -\alpha }{3}$$ (33)

$$p_{H}^{*}=\frac{2(\beta -\alpha )}{3}$$ (34)

quantities:

$$x_{L}^{*}=\frac{1}{3}$$ (35)

$$x_{H}^{*}=\frac{2}{3}$$ (36)

and profits:

$$\Pi_{L}^{*}=p_{L}^{*}x_{L}^{*}=\frac{\beta -\alpha }{9}$$ (37)

$$\Pi_{H}^{*}=p_{H}^{*}x_{H}^{*}=\frac{4(\beta -\alpha )}{9}$$ (38)

In a covered market, the more differentiated are the low- and high-quality products, the greater are the profits of the low- and high-quality firms, that is, $\frac{\partial \Pi_{H}}{\partial \beta }>0,\frac{\partial \Pi_{L}}{\partial \beta }>0,\frac{\partial \Pi_{H}}{\partial \alpha }<0$ and $\frac{\partial \Pi_{L}}{\partial \alpha }<0$.

### Stage 1: Excess information decisions in a covered market

As discussed and analyzed earlier, the consumer reaction to excess information can be positive or negative affecting the consumer valuation of the relevant product(s), and the equilibrium prices, quantities and firm profits. In this context, based on the impacts of the strategy on the profits of each firm, there are two possible scenarios that can emerge and are discussed below.

### Case A: When the consumer reaction to excess information is positive

When, by adopting the strategy, the firms can increase the consumer valuation of their products, the dominant strategy of the low-quality firm is to not adopt the strategy (as $\frac{\partial \Pi_{L}}{\partial \alpha }<0$ due to increased *α* reducing the product differentiation). On the other hand, the dominant strategy of the high-quality firm is to adopt the strategy (as $\frac{\partial \Pi_{H}}{\partial \beta }>0$ due to increased *β* increasing product differentiation), resulting in the strategy profile (*E*, *N*) being the unique Nash equilibrium.

**Result 9:** When the consumer reaction to excess information is positive and firms compete in a covered market, only the high-quality firm adopts the excess information strategy.

### Case B: When the consumer reaction to excess information is negative

When the consumer reaction to excess information is negative, the dominant strategy of the high-quality firm is to not adopt the strategy (as $\frac{\partial \Pi_{H}}{\partial \beta }>0$), while the dominant strategy of the low-quality firm is to adopt the strategy (as $\frac{\partial \Pi_{L}}{\partial \alpha }<0$). Thus, in this case (*N*, *E*) is the unique Nash equilibrium.

**Result 10:** When the consumer reaction to excess information is negative and firms compete in a covered market, only the low-quality firm adopts the excess information strategy.

### Economic impacts of excess information in a covered market

In this section we analyze how the equilibrium prices and quantities of the low- and high-quality products, firm profits and consumer welfare change under the two cases examined above.

### Case A: Only the high-quality firm adopts the excess information strategy (the consumer reaction to excess information is positive)

When only the high-quality firm adopts the strategy in a covered market, the equilibrium prices of the low- and high-quality products in the second stage of the game are given by:

$${p_{L}^{\prime }}^{*}=\frac{\beta^{\prime }-\alpha }{3}=\frac{\beta +\varepsilon -\alpha }{3}$$ (39)

$${p_{H}^{\prime }}^{*}=\frac{2(\beta^{\prime }-\alpha )}{3}=\frac{2(\beta +\varepsilon -\alpha )}{3}$$ (40)

The equilibrium prices of both low- and high-quality products are higher than those under no excess information with the price increase being higher for the high-quality product.

The equilibrium quantities of low- and high-quality products are:

$${x_{L}^{\prime }}^{*}=\frac{1}{3}$$ (41)

$${x_{H}^{\prime }}^{*}=\frac{2}{3}$$ (42)

and the profits of the low- and high-quality firms are given by:

$${\Pi_{L}^{\prime }}^{*}={p_{L}^{\prime }}^{*}{x_{L}^{\prime }}^{*}=\frac{\beta^{\prime }-\alpha }{9}=\frac{\beta +\varepsilon -\alpha }{9}$$ (43)

$${\Pi_{H}^{\prime }}^{*}={p_{H}^{\prime }}^{*}{x_{H}^{\prime }}^{*}=\frac{4(\beta^{\prime }-\alpha )}{9}=\frac{4(\beta +\varepsilon -\alpha )}{9}$$ (44)

As profits of both low- and high-quality firms increase with an increase in *β*, the profits of the firms are higher when only the high-quality firm adopts the strategy of excess information than under the no excess information case.

**Fig 6:**
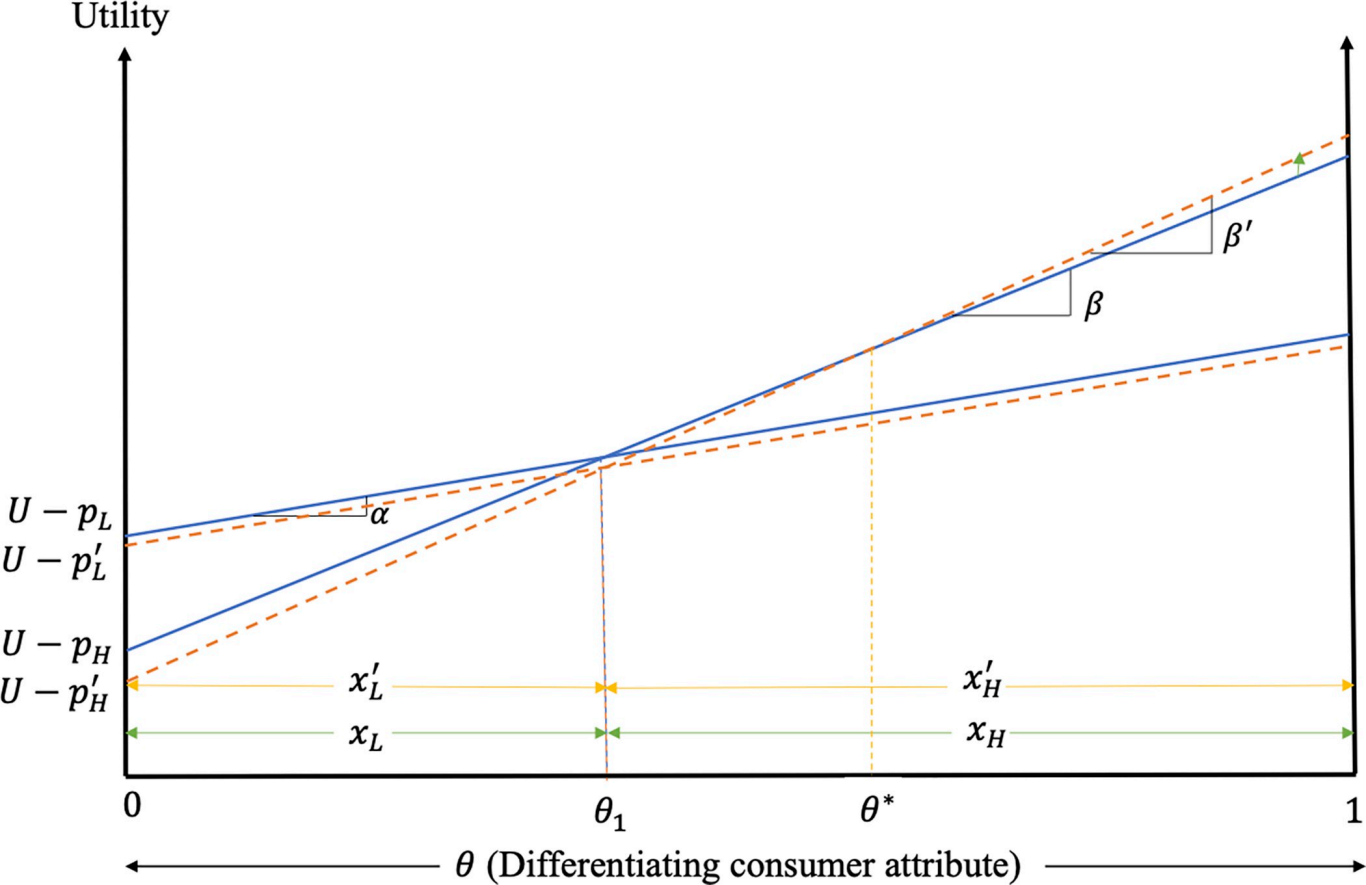
Impacts of excess information on consumer decisions and welfare when only the high-quality firm provides excess information in a covered market.

Fig 6 graphs the impacts of excess information on consumer decisions and welfare when only the high-quality firm adopts the strategy.

Despite the increased consumer valuation of the high-quality product in this case, the greater increase in the price of this product prevents the consumers of the low-quality product with stronger preference for quality from moving to high-quality product, keeping the market shares for both products the same as those under no excess information. The consumers of low-quality product experience welfare losses due to the higher price of the product, and this change in the welfare is given by $\Delta W_{L}^{C}=\int_{0}^{\theta_{1}}(U_{L}^{\prime }-U_{L})d\theta$.

Among the consumers of high-quality product, those with weaker preference for quality (i.e., *θ*∈[*θ*_1_, θ*)) experience welfare losses, while those with stronger preference for quality (i.e., *θ*∈(θ*, 1]) realize welfare gains. The change in the welfare of the consumers of high-quality product is given by $\Delta W_{H}^{C}=\int_{\theta_{1}}^{1}(U_{H}^{\prime }-U_{H})d\theta$.

**Result 11:** In a covered market, when the consumer reaction to excess information is positive, only the high-quality firm adopts the strategy. The equilibrium prices of low- and high-quality products increase due to the increase in the degree of differentiation between the two products, while the market shares of these products remain unchanged. The profits of both low- and high-quality firms are higher than under the no excess information case. Consumers of the low-quality product and consumers of the high-quality product with weaker preference for quality experience welfare losses due to increased prices, while the consumers of high-quality product with stronger preference for quality experience welfare gains due to the increased valuation of the high-quality product.

### Case B: Only the low-quality firm adopts the excess information strategy (the consumer reaction to excess information is negative)

When only the low-quality firm adopts the strategy in a covered market, the equilibrium prices of the low- and high-quality products are given by:

$${p_{L}^{\prime }}^{*}=\frac{\beta -\alpha^{\prime \prime }}{3}=\frac{\beta -\alpha +\delta }{3}$$ (45)

$${p_{H}^{\prime }}^{*}=\frac{2(\beta -\alpha^{\prime \prime })}{3}=\frac{2(\beta -\alpha +\delta )}{3}$$ (46)

As *α*′′<*α*, the equilibrium prices are higher than those under no excess information with the price increase being greater for the high-quality product. The equilibrium quantities remain unchanged at:

$${x_{L}^{\prime }}^{*}=\frac{1}{3}$$ (47)

$${x_{H}^{\prime }}^{*}=\frac{2}{3}$$ (48)

while the profits of the low- and high-quality firms are given by:

$${\Pi_{L}^{\prime }}^{*}={p_{L}^{\prime }}^{*}{x_{L}^{\prime }}^{*}=\frac{\beta -\alpha^{\prime \prime }}{9}=\frac{\beta -\alpha +\delta }{9}$$ (49)

$${\Pi_{H}^{\prime }}^{*}={p_{H}^{\prime }}^{*}{x_{H}^{\prime }}^{*}=\frac{4(\beta -\alpha^{\prime \prime })}{9}=\frac{4(\beta -\alpha +\delta )}{9}$$ (50)

As profits of both low- and high-quality firms increase with an increase in the degree of product differentiation, the profits of the firms are higher when only the low-quality firm adopts the strategy of excess information relative to the no excess information case.

**Fig 7:**
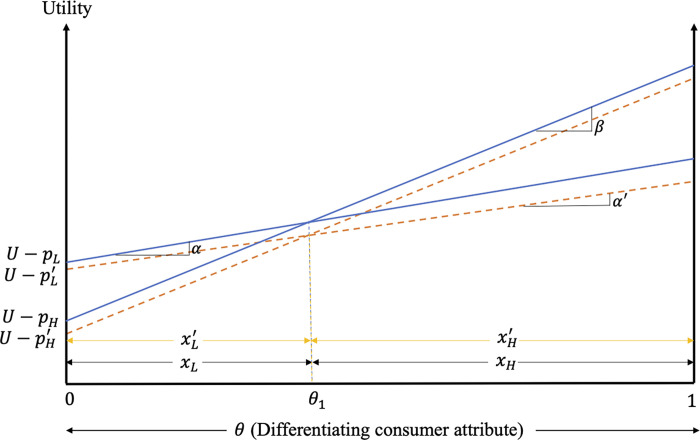
Impacts of excess information on consumer decisions and welfare when only the low-quality firm provides excess information in a covered market.

Fig 7 graphs the impacts of excess information on consumer decisions and welfare when only the low-quality firm adopts the strategy in a covered market.

Fig 7 shows that, even though the consumer valuation of low-quality product is lower than the benchmark case, the greater increase in the price of the high-quality product prevents the consumers of low-quality product with stronger preference for quality to move to the high-quality product, while consumers of the high-quality product have no incentives to switch to the low-quality product due to lower consumer valuation of the product, keeping the market shares the same as under the no excess information case. Consumers of the low-quality product experience welfare losses due to lower consumer valuation and higher price of the product, with the change in their welfare given by $\Delta W_{L}^{C}=\int_{0}^{\theta_{1}}(U_{L}^{\prime }-U_{L})d\theta$. The consumers of the high-quality product also experience welfare losses in this case due to the higher price of the product, with the change in their welfare given by $\Delta W_{H}^{C}=\int_{\theta_{1}}^{1}(U_{H}^{\prime }-U_{H})d\theta$.

**Result 12:** In a covered market, when the consumer reaction to excess information is negative, only the low-quality firm adopts the excess information strategy. In this case, the equilibrium prices of low- and high-quality products increase due to the increase in the degree of product differentiation, while the market shares remain unchanged. Both firms earn higher profits, while consumers of low- and high-quality products experience welfare losses due to higher product prices.

### Concluding remarks

The rise in the consumer demands for food products with various credence attributes has provided food companies with incentives to make related claims on their product labels. Although information on food product labels is usually helpful for consumers of goods with unobservable (credence or experience) attributes, some food claims, though not false, can be redundant and potentially misleading.

While increased profits appear to be the key motivating factor of this excess information strategy, not all firms adopt the strategy (and not all relevant products in the market bear labels with such claims). Previous literature has focused on the consumer reaction to different food claims and the reasons behind this reaction. To our knowledge, the causes of different firms adopting this excess information strategy and the economic consequences of the strategy have not been considered previously. This paper addresses this issue and systematically analyzes the economic causes and consequences of excess information.

To determine the causes and market and welfare consequences of excess information, the paper develops novel, empirically-relevant game-theoretic models of heterogeneous firms and consumers in vertically differentiated markets with asymmetric information. The analysis reveals that the firm incentives to adopt the strategy, the Nash equilibrium configuration of firms adopting the strategy, and the market and welfare impacts of excess information are case-specific and dependent on the firms’ product quality, the degree of product differentiation, the consumer reaction to excess information, and whether the market is covered or not.

The case-specific nature of the results is highlighted by the impacts of excess information on different firms and consumers in an uncovered market, where, under positive consumer reaction to excess information, the adoption of the strategy by both low- and high-quality firms benefits the consumers of the low- and high-quality products the most. When the consumer reaction to excess information is negative, both consumer groups prefer that firms do not adopt the strategy. The high-quality firm profits are the highest when it is the only one adopting the strategy, while the low-quality firm’s profits are the highest when the consumer reaction to excess information is positive and both firms adopt the strategy.

The determination of the key factors affecting the causes and market and welfare consequences of excess information can inform the analysis of specific cases of excess information in food claims. Thus, in addition to providing important new insights on the economic causes and consequences of excess information, our analysis can provide a valuable theoretical grounding for empirical studies of excess information provision in quality-differentiated food product markets.

## Supporting information

S1 Appendix Derivation of equilibrium strategies in extensive excess information games. (DOCX)
