# Supplementary material for: Causes and consequences of fake transparency/excess information in food claims
Source: PLoS One. 2022 Dec 8;17(12):e0275800. doi: 10.1371/journal.pone.0275800 (PMC9731450; doi:10.1371/journal.pone.0275800)
Supplement: S1 Appendix — (DOCX) [file pone.0275800.s001.docx]

**Appendix**

**Derivation of equilibrium strategies in extensive excess information games**

In this section we derive the equilibrium strategy profiles under different scenarios on the consumer reaction to excess information and degree of differentiation between the low- and high-quality products analyzed earlier, when the firms make their excess information decisions in the first stage of the game sequentially (rather than simultaneously). The subsequent price competition and consumer decisions are similar to those analyzed at the main part of this article.

We begin by considering the case where the high-quality firm moves first and makes the decision regarding adopting (or not) the excess information strategy, followed by the low-quality firm, which observes the decision of its rival prior to making its own decision regarding the strategy. The game in the first stage of the strategic interaction between the low- and high-quality firms can be represented as shown in the game tree in Fig A1.

**Fig A1. Extensive form representation of the excess information game where the high-quality firm moves first.**

Assuming complete information, we can find the subgame perfect equilibrium of this sequential game as the set of strategies that induces a Nash equilibrium in every subgame of the original game shown above. We can derive the subgame perfect equilibrium (SPE) of the game under the different scenarios using backward induction. It turns out that, due to the dominance of the firms’ strategies, the equilibrium strategies of the low- and high-quality firms (and the market and welfare impacts of excess information) are similar to those in the strategic excess information games analyzed in the main part of this research.

***Case i: When the consumer reaction to excess information is positive and the low- and high-***

***quality products are highly differentiated***

When the consumer reaction to excess information is positive and the low- and high-quality products are highly differentiated (i.e., $\alpha<\frac{4}{7}\beta$), the low-quality firm’s profits when it adopts the strategy are higher than when it does not as $\frac{\partial\Pi_{L}}{\partial\alpha}>0$ for any strategy of the high-quality firm (resulting in $\Pi_{L}^{E,E}>\Pi_{L}^{E,N}$ on the left node and $\Pi_{L}^{N,E}>\Pi_{L}^{N,N}$ on the right node). Similarly, no matter what the low-quality firm’s response is, the optimal strategy of the high-quality firm is to adopt the strategy as $\frac{\partial\Pi_{H}}{\partial\beta}>0$. Therefore, in this case $(E,(E,E))$ is the SPE of the extensive excess information game.

***Case ii: When the consumer reaction to excess information is positive and the low- and high-***

***quality products are not highly differentiated***

When the consumer reaction to excess information is positive and the difference in quality of the low- and high-quality products is relatively small (i.e., $\alpha>\frac{4}{7}\beta$), the low-quality firm’s profits are higher when it does not adopt the strategy as, under this scenario, $\frac{\partial\Pi_{L}}{\partial\alpha}<0$ (resulting in $\Pi_{L}^{E,N}>\Pi_{L}^{E,E}$ on the left node and $\Pi_{L}^{N,E}>\Pi_{L}^{N,N}$ on the right node). On the other hand, the high-quality firm’s best option, in this case, is to adopt the strategy as $\frac{\partial\Pi_{H}}{\partial\beta}>0$ (and, thus, $\Pi_{H}^{E,N}>\Pi_{H}^{N,E}$). Thus, when the consumer valuation of excess information is positive and the low and high-quality products are not highly differentiated, $(E,(N,E))$ is the SPE of the excess information game.

***Case iii: When the consumer reaction to excess information is negative and the low- and high-***

***quality products are highly differentiated***

When the consumer reaction to excess information is negative and the low- and high-quality products are highly differentiated, the low-quality firm will not adopt the strategy irrespective of the high-quality firm’s strategy as, under this scenario, $\frac{\partial\Pi_{L}}{\partial\alpha}<0$ (resulting in $\Pi_{L}^{E,N}>\Pi_{L}^{E,E}$ on the left node and $\Pi_{L}^{N,N}>\Pi_{L}^{N,E}$ on the right node). The best strategy for the high-quality firm, in this case, is to not introduce excess information on their product either as a reduction in $\beta$ decreases the firm’s profits (making $\Pi_{H}^{N,N}>\Pi_{H}^{E,N}$). Therefore, under this scenario $(N,(N,N))$ is the SPE of the excess information game.

***Case iv: When the consumer reaction to excess information is negative and the low- and high-***

***quality products are not highly differentiated***

When the consumer reaction to excess information is negative and the difference in quality of the low- and high-quality products is relatively small (i.e.,$\alpha>\frac{4}{7}\beta$), then the low-quality firm finds it optimal to adopt the strategy as, by reducing $\alpha$, the low-quality firm can increase its profits (resulting in $\Pi_{L}^{E,E}>\Pi_{L}^{E,N}$ on the left node and $\Pi_{L}^{N,E}>\Pi_{L}^{N,N}$on the right node). The optimal choice of the high-quality firm is to not adopt the strategy as the profits of the high-quality firm fall with a reduction in $\beta$ (resulting in $\Pi_{H}^{N,E}>\Pi_{H}^{E,E}$). Therefore, when the difference in the quality of the two products is small and the consumer reaction to excess information is negative, $(N,(E,E))$ is the SPE of the excess information game.

Similarly, we can derive the SPEs of the extensive excess information game where the low-quality firm moves first and decides on its excess information strategy and is followed by the high-quality firm, which makes its decision about adopting the excess information strategy after observing the move of the low-quality firm (see Fig A2).

**Fig A2. Extensive form of the excess information game where the low-quality firm moves first**

***Case v: When the consumer reaction to excess information is positive and the low- and high-***

***quality products are highly differentiated***

When the consumer reaction to excess information is positive, the high-quality firm chooses to adopt the strategy irrespective of the low-quality firm’s move as $\frac{\partial\Pi_{H}}{\partial\beta}>0$ (resulting in $\Pi_{H}^{E,E}>\Pi_{H}^{E,N}$ on the left node and $\Pi_{H}^{N,E}>\Pi_{H}^{N,N}$ on the right node). When the low- and high-quality products are highly differentiated (i.e., $\alpha<\frac{4}{7}\beta$), the low-quality firm’s profits increase with $\alpha$ making the provision of excess information the optimal strategy of the low-quality firm. Thus, in this case, $(E,(E,E))$ is the SPE of the excess information game.

***Case vi: When the consumer reaction to excess information is positive and the low- and high-***

***quality products are not highly differentiated***

When the consumer reaction to excess information is positive, the high-quality firm will always find it optimal to adopt the strategy as $\frac{\partial\Pi_{H}}{\partial\beta}>0$ (resulting in $\Pi_{H}^{E,E}>\Pi_{H}^{E,N}$ on the left node and $\Pi_{H}^{N,E}>\Pi_{H}^{N,N}$ on the right node). However, when $\alpha>\frac{4}{7}\beta$, then $\frac{\partial\Pi_{L}}{\partial\alpha}<0$ resulting in $\Pi_{L}^{N,E}>\Pi_{L}^{E,E}$ and the low-quality firm not adopting the excess information strategy. Therefore, in this case $(N,(E,E))$ is the SPE of this game.

***Case vii: When the consumer reaction to excess information is negative and the low- and***

***high-quality products are highly differentiated***

When the consumer reaction to excess information is negative and the difference in the quality of low- and high-quality products is high, then the high-quality firm will always find it optimal to not adopt the strategy as $\frac{\partial\Pi_{H}}{\partial\beta}>0$ (resulting in $\Pi_{H}^{E,N}>\Pi_{H}^{E,E}$ on the left node and $\Pi_{H}^{N,N}>\Pi_{H}^{N,E}$ on the right node). The low-quality firm will also not adopt the strategy as $\frac{\partial\Pi_{L}}{\partial\alpha}>0$ (resulting in $\Pi_{L}^{N,N}>\Pi_{L}^{E,N}$). Thus, in this case, $(N,(N,N))$ is the SPE of the excess information game.

***Case viii: When the consumer reaction to excess information is negative and the low- and***

***high-quality products are not highly differentiated***

When the consumer reaction to excess information is negative and the quality difference between the two products is relatively low (i.e., $\alpha>\frac{4}{7}\beta$) the high-quality firm will not find it optimal to adopt the strategy as $\frac{\partial\Pi_{H}}{\partial\beta}>0$ (resulting in $\Pi_{H}^{E,N}>\Pi_{H}^{E,E}$ on the left node and $\Pi_{H}^{N,N}>\Pi_{H}^{N,E}$ on the right node). However, when $\alpha>\frac{4}{7}\beta$, then $\frac{\partial\Pi_{L}}{\partial\alpha}<0$ and the low-quality firm will adopt the strategy as $\Pi_{H}^{E,N}>\Pi_{H}^{N,N}$. Under this scenario, $(E,(N,N))$ is the SPE of the excess information game.
